# Letter regarding “The role of reviewers in the era of systematic reviews and meta-analysis: A practical guide for researchers”

**DOI:** 10.17305/bb.2026.14264

**Published:** 2026-04-13

**Authors:** Himel Mondal

**Affiliations:** 1 Department of Physiology, All India Institute of Medical Sciences, Deoghar, Jharkhand, India

I read with great interest the recent article on the role of reviewers in systematic reviews and meta-analyses (SRMAs). The paper provides a clear and practical guide for assessing methodological quality [1]. However, three additional concerns warrant attention in the current peer review landscape.

I concur with the authors that the use of artificial intelligence without appropriate human oversight poses potential risks. While artificial intelligence *(*AI)-generated text may appear fluent and convincing, it can contain errors, fabricated information, or misleading interpretations [2]. It is crucial, however, to encourage authors to utilize AI tools for enhancing language clarity, grammar correction, and overall readability. Nonetheless, the dilemma arises when both AI-generated and AI-edited texts yield similar results in AI assessments [3]. Consequently, reviewers must depend on authors’ disclosures regarding the extent of AI involvement. The primary issue lies not in the use of AI itself, but in the lack of transparency and critical human verification.

An overreliance on text similarity reports can lead to unintended consequences. When strict similarity thresholds are enforced, authors may excessively paraphrase standard methodological descriptions to lower the overall similarity index. This often results in awkward or convoluted phrasing that diminishes clarity rather than enhancing originality [4]. In the context of SRMAs, where methodological standards are inherently uniform, some degree of textual similarity is inevitable. Therefore, reviewers should interpret the overall similarity index with caution.

The task of reference verification has increasingly burdened reviewers, particularly given the rise of AI-generated or inaccurate citations. Reviewers attempting to verify multiple references often encounter practical barriers, including repeated security checks such as completely automated public Turing test to tell computers and humans apart (CAPTCHA) or Cloudflare verification (Figure 1). This requirement forces reviewers to demonstrate their humanity to a machine, consuming valuable time [5]. Such delays significantly impede the review process and contribute to reviewer fatigue. Consequently, thorough reference checking becomes challenging, despite its critical role in ensuring the reliability of scientific work.

**Figure 1.**
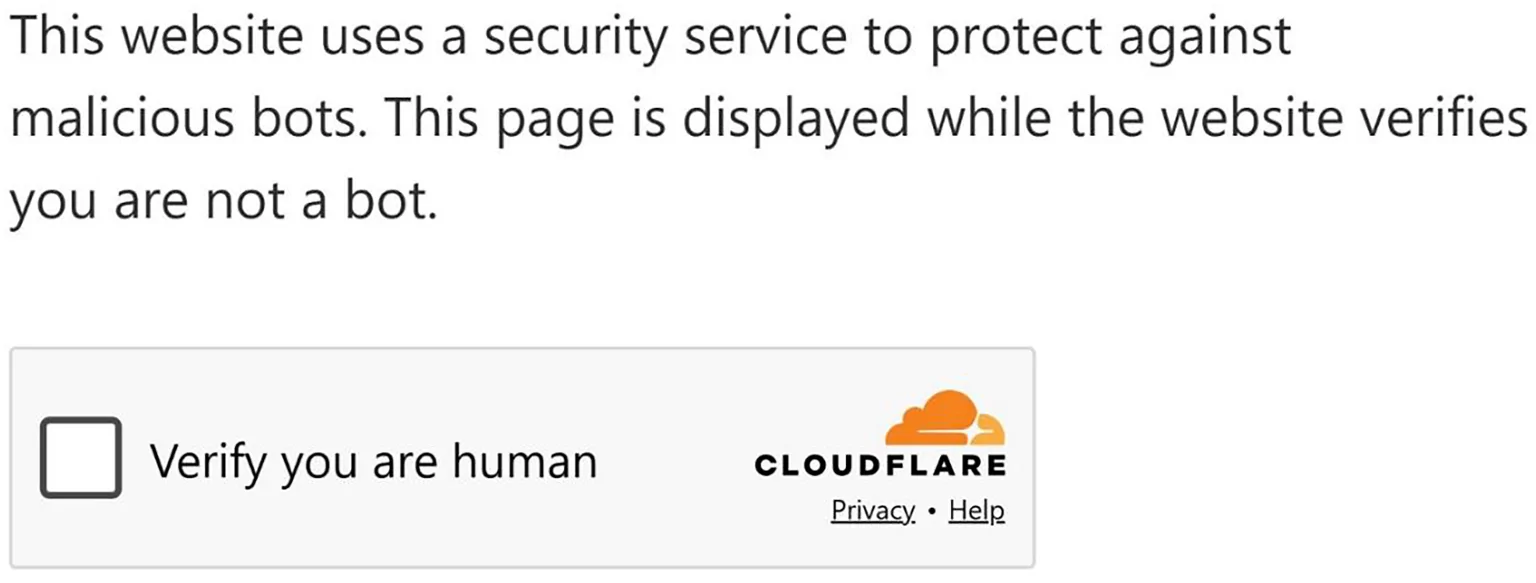
**Cloudflare human-verification screen encountered while accessing a cited journal article during reference checking.** This browser-based security prompt illustrates a practical barrier to reference verification during peer review, as repeated access interruptions can delay reviewers and increase workload and fatigue.

A practical solution necessitates coordinated efforts from journals, authors, and reviewers. Journals should enhance initial editorial screening by employing reliable plagiarism detection and automated reference-checking tools prior to peer review. Clear guidelines for the Methods section of an SRMA should be established to minimize unnecessary paraphrasing. Additionally, publishers should improve access systems to reduce interruptions from repeated CAPTCHA or security checks during reference verification.

Strengthening editorial policies, enhancing technological support, and acknowledging the practical limitations faced by reviewers will be essential to uphold the integrity and efficiency of peer review in this rapidly evolving landscape.
